# Light and the laboratory mouse

**DOI:** 10.1016/j.jneumeth.2017.04.007

**Published:** 2018-04-15

**Authors:** Stuart N. Peirson, Laurence A. Brown, Carina A. Pothecary, Lindsay A. Benson, Angus S. Fisk

**Affiliations:** Sleep and Circadian Neuroscience Institute (SCNi), Nuffield Department of Clinical Neurosciences, University of Oxford, Oxford Molecular Pathology Institute, Dunn School of Pathology, South Parks Road, Oxford, United Kingdom

**Keywords:** Circadian, Retina, Wavelength, Welfare

## Abstract

Light exerts widespread effects on physiology and behaviour. As well as the widely-appreciated role of light in vision, light also plays a critical role in many non-visual responses, including regulating circadian rhythms, sleep, pupil constriction, heart rate, hormone release and learning and memory. In mammals, responses to light are all mediated via retinal photoreceptors, including the classical rods and cones involved in vision as well as the recently identified melanopsin-expressing photoreceptive retinal ganglion cells (pRGCs). Understanding the effects of light on the laboratory mouse therefore depends upon an appreciation of the physiology of these retinal photoreceptors, including their differing sens itivities to absolute light levels and wavelengths. The signals from these photoreceptors are often integrated, with different responses involving distinct retinal projections, making generalisations challenging. Furthermore, many commonly used laboratory mouse strains carry mutations that affect visual or non-visual physiology, ranging from inherited retinal degeneration to genetic differences in sleep and circadian rhythms. Here we provide an overview of the visual and non-visual systems before discussing practical considerations for the use of light for researchers and animal facility staff working with laboratory mice.

## Highlights

• Light exerts widespread effects on physiology and behaviour in laboratory mice.
• These effects are mediated by both visual and non-visual photoreceptor systems.
• Many commonly used laboratory mouse strains carry mutations that affect these systems.
• Here we provide practical considerations for the use of light for researchers working with laboratory mice.

## 1 Introduction

The rotation of the earth on its axis produces a daily variation in the light environment under which all life has evolved. Whilst light is typically defined as the component of the electromagnetic spectrum used for vision, ranging from 380 to 780 nm, this anthropocentric definition overlooks the fact that different organisms possess greatly differing photoreceptor complements and as such different spectral sensitivities (Sliney, 2016). In mammals, detection of light by retinal photoreceptors forms the first step in the visual pathway, which culminates in a representation of the external environment at the level of the visual cortex. However, in addition to these classical visual pathways with which many researchers are familiar, light also exerts profound effects on a physiology and behaviour. Such responses do not involve image-forming pathways, instead relying upon detection of changes in the environmental irradiance (brightness). These non-visual responses include entrainment of circadian rhythms, regulation of sleep, pupillary constriction, regulation of hormones as well as modulation of cognitive processes.

Mice are the organism of choice in biomedical research due to their small size, rapid reproductive cycles, coupled with the availability of extensive genomic information and well-established transgenic resources. The mouse visual system has been studied extensively and continues to provide a valuable model for basic neuroscience as well as the study of human disease. In addition, most of the major advances in our understanding of non-visual responses to light over the last two decades has been based upon studies in the mouse. In this review, we will summarise the visual and non-visual systems of the laboratory mouse, including the retinal photoreceptors that mediate these responses, differences in common laboratory strains and practical recommendations for rodent husbandry and research.

## 2 The mouse visual system

As a nocturnal rodent, mice are often not considered to depend upon their visual system, instead relying on olfactory, tactile and auditory cues to locate food, interact with conspecifics and avoid predation. Mouse visual acuity is poor, estimated to be equivalent to 20/2000 human vision, which would qualify them as legally blind (Baker, 2013). Whilst there are clearly limits to the visual capabilities of mice, numerous studies have shown that mice display visually-guided behaviours and a wide range of specialised assays have been developed for the study of mouse vision (Prusky and Douglas, 2008).The mouse visual system serves a number of very different functions ranging from simple object detection to pattern discrimination, movement detection, visual acuity and detecting changes in environmental light levels. As such, there is no one test of vision in mice and different assays provide information relating to different aspects of visual function (Pinto and Enroth-Cugell, 2000). For example, the visual cliff provides a simple test to assess visually-guided behaviour (Crawley, 1999), whereas more complex tasks such as the visual water maze and optomotor test enable visual acuity and contrast sensitivity to be determined (Douglas et al., 2005, Prusky and Douglas, 2008). Whilst the pupillary light response provides an overall measure of retinal sensitivity to light (Lucas et al., 2001), such responses can be retained in the absence of image-forming vision. By contrast, electroretinography (ERG) is the gold standard measure of retinal function, but does not provide any information relating to how this information is subsequently processed by the brain (Cameron et al., 2008).

### 2.1 Anatomy

**Fig. 1:**
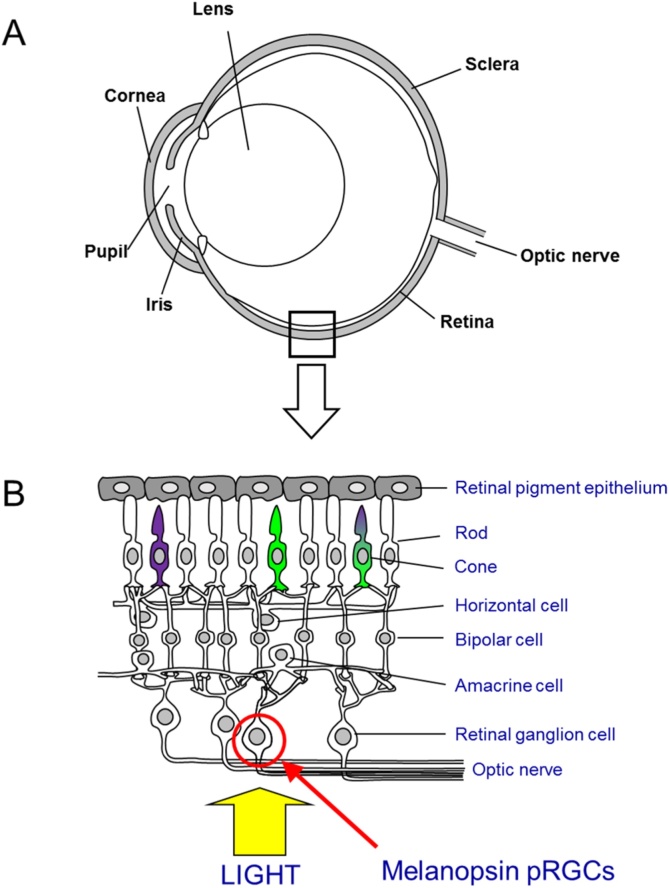
The mouse eye and retina. A) The mouse eye is similar in structure to that of most other vertebrates, although the lens is relatively larger. B) The retina is a layered structure and light must pass through the inner retinal layers to reach the light sensitive photoreceptors in the outer retina. The retina contains two classes of visual photoreceptor, rods which mediate low light (scotopic) vision and cones which mediate bright light (photopic) vision and provide colour vision. Mice have two cone visual pigments, an ultraviolet light sensitive (UVS) opsin and a middle-wavelength sensitive (MWS) opsin. However, in 95% of cones, these opsins are co-expressed. In addition to the rods and cones, a subset of melanopsin-expressing photosensitive retinal ganglion cells (pRGCs) have recently been identified, mediating many non-visual responses to light.

Although laboratory mice are exposed to a very different visual environment than their ancestors, studies on the eyes of inbred strains have shown no differences in retinal anatomy and visual pigment expression compared to wild mice (Shupe et al., 2006). Anatomically, the mouse eye is broadly comparable to that of most other vertebrate species, with a cornea and lens that refract light to form an image on the light sensitive retina (Fig. 1A). The eye has an axial length of 3.4 mm (Leamey et al., 2008, Remtulla and Hallett, 1985), and this small size necessitates a large lens accounting for 60% of the axial length (Remtulla and Hallett, 1985). Like many other mammalian species, this lens transmits ultraviolet light, necessary for the ultraviolet sensitive photoreceptors found in the mouse retina (Douglas and Jeffery, 2014). As in all vertebrates, the retina is a highly stratified structure (Fig. 1B), comprising the outer retina containing the photoreceptor cell bodies and outer segments, and the inner retina − consisting of bipolar cells, amacrine cells and the retinal ganglion cells (RGCs). The retina is around 200 μm thick, with photoreceptors accounting for around half of this. As such, in strains with retinal degeneration (see below), the retina may be noticeably thinner when extracted or viewed in histopathology sections. Bipolar cells convey signals from the photoreceptors to the amacrine and ganglion cells, though in the case of rods, this is actually via AII amacrine cells. The RGCs provide the primary output neurons of the retina, their axons forming the optic nerve that projects to the brain. In addition to the neurons of the retina, the retinal pigment epithelium (RPE) lies adjacent to the photoreceptor layer and provides the site of photopigment regeneration via the visual cycle (Thompson and Gal, 2003, Wright et al., 2015), and Műller cells span the entire thickness of the retina providing glial support (Bringmann et al., 2006).

### 2.2 Photoreceptors

The retinal photoreceptor layer contains rod and cone photoreceptors which mediate scotopic (low light) and photopic (bright light) vision, respectively. Like many mammals, and especially nocturnal rodents, the retina is rod-dominated with around 6.4 million rods, accounting for ∼97% of photoreceptors (Leamey et al., 2008). By contrast, there are only around 200,000 cones, accounting for just 3% of the photoreceptors (Carter-Dawson and LaVail, 1979, Jeon et al., 1998). The mouse retina does not possess a *fovea centralis*, the central region of the primate retina with the highest cone density which lacks rods and other retinal neurons. However, the rod and cone density peaks in the *area centralis* and decreases peripherally. The peak rod density is around 100,000/mm^2^ whereas the peak cone density is around 16,000/mm^2^. As such, the photoreceptor density is comparable to that of the cat or macaque peripheral retina (Leamey et al., 2008).

The photoreceptor outer segment is where the light sensitive visual pigments are expressed − transmembrane proteins consisting of an opsin protein bound to a light sensitive vitamin-A based chromophore − 11-*cis* retinal. Absorption of light is the first step in vision, leading to isomerisation of the 11-*cis* retinal into the all-*trans* state. This results in a change in the conformational state of the opsin protein, allowing activation of the G-protein transducin. This in turn leads to activation of phosphodiesterase which hydrolyses cGMP into GMP, leading to closure of cyclic nucleotide gated ion channels and a hyperpolarisation of the photoreceptor cell. As such, photoreceptor cells are depolarised in the dark and constitutively release glutamate, effectively reducing their output signal in response to light (Arshavsky et al., 2002, Fain et al., 2010, Lamb, 2009).

### 2.3 Visual pigments

**Fig. 2:**
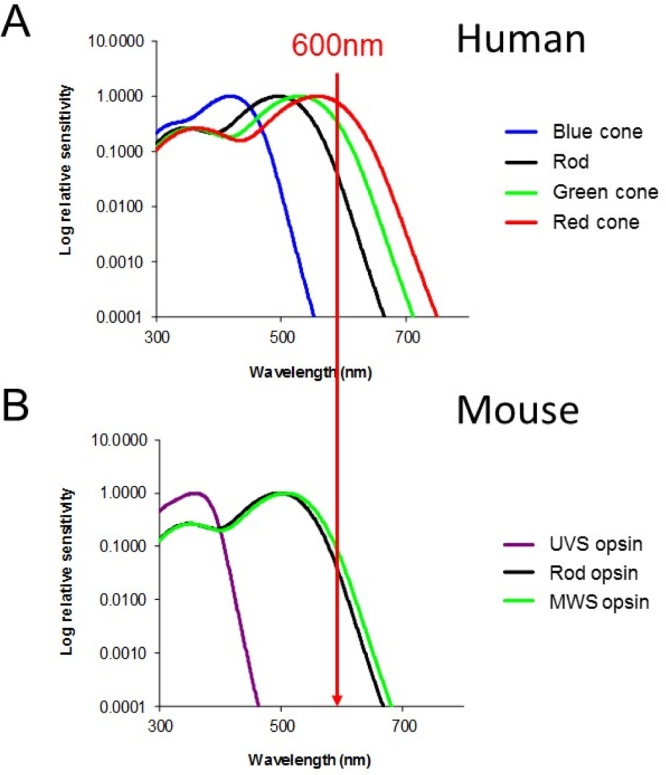
Spectral sensitivity of human and mouse visual pigments. The human and mouse retina contain a different complement of light-sensitive visual pigments. A) The human retina contains rods and three cone classes, maximally sensitive to red, green and blue light. B) By contrast, the mouse retina is rod dominated (97% of photoreceptors) and contains cone opsins maximally sensitive to ultraviolet and green light. As a result, mice are relatively less sensitive to long wavelength light. For example, at 600 nm (red light), the human visual system is 12 times more sensitive than the mouse visual system. As such, whilst mice are less sensitive to red light, care must be taken to ensure that such stimuli are as dim as possible to allow researchers and animal facility staff to operate. (For interpretation of the references to colour in this figure legend, the reader is referred to the web version of this article.)

The mouse retina contains three visual pigments − a rod opsin with a peak sensitivity (λ_max_) at 498 nm and middle wavelength sensitive (MWS) and ultraviolet sensitive (UVS) cone opsins with λ_max_ 508 nm and ∼360 nm, respectively (Bridges, 1959, Jacobs et al., 1991, Sun et al., 1997). However, unlike most mammalian species, these cone opsins are co-expressed in a dorsal-ventral gradient across the mouse retina, with highest UVS opsin in the ventral retina and highest MWS opsin in the dorsal retina. Whilst 95% of cones co-express UVS and MWS opsins, a subset of cones express just the UVS opsin and are found distributed across the whole retina (Applebury et al., 2000, Haverkamp et al., 2005, Nikonov et al., 2006, Rohlich et al., 1994). Due to this UVS visual pigment, mice show an increased sensitivity to UV light in comparison with humans (Jacobs et al., 1991, Jacobs et al., 2004). However, due to the lack of a long wavelength sensitive opsin, mice are less sensitive to longer wavelength light (Fig. 2). For example, given a red light stimulus at 600 nm, based on the visual pigments of the human and mouse retina, the mouse eye will be 12 time less sensitive. However, this does not mean that mice cannot detect such light, and if sufficiently bright, mice are certainly capable of responding to such long wavelength stimuli.

## 3 Circadian photoreception

In constant conditions virtually all organisms continue to exhibit rhythms in physiology and behaviour with a period around 24 h. These so-called circadian rhythms (from the Latin for ‘around a day’) are the product of an internal biological clock, and enable organisms to anticipate rhythmic changes in their environment, preparing physiology and behaviour in readiness to exploit these predictable variations, rather than simply responding to them. The circadian clock in mice is typically ∼23.5 h, so in constant darkness, mice advance their activity onset every day (Jud et al., 2005). In mammals, light provides the primary time cue to synchronise – or entrain – the circadian system to the external environment. In mammals, the photoreceptors mediating this process are exclusively ocular, and enucleation or loss of the eye blocks all responses to light (Foster et al., 1991, Nelson and Zucker, 1981).

The role of the eye in circadian photoreception led researchers to ask whether the rod or cone photoreceptors mediated these responses. In the early 1990s, researchers studied mice carrying the *rd/rd* mutation (*Pde6b^rd1^*), in which rods degenerate and cones are also subsequently lost (Pittler and Baehr, 1991). Remarkably, despite being visually blind, these animals retained their ability to shift their circadian clock in response to light (Foster et al., 1991). Subsequent studies showed that the spectral sensitivity of these responses differed from that of the rods and cones (Provencio and Foster, 1995, Yoshimura and Ebihara, 1996). However, as a small proportion of cones may be retained in the *rd/rd* retina, so to address this issue, transgenic mice lacking all rods and cones were subsequently generated. Both circadian phase-shifting and suppression of pineal melatonin responses to light were preserved in these animals (Freedman et al., 1999, Lucas et al., 1999), suggesting that a third class of photoreceptor existed within the inner retina.

### 3.1 Melanopsin pRGCs

**Fig. 3:**
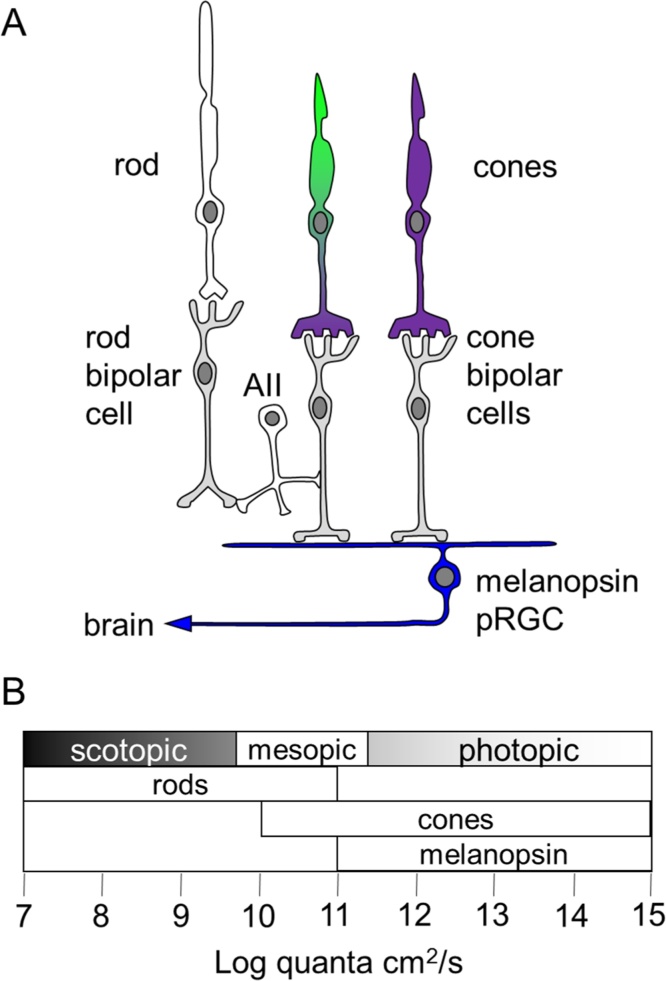
Interaction between rods, cones and melanopsin pRGCs. A) Rods and cones provide input to melanopsin pRGCs. Note that most cones co-express UVS and MWS opsin. Rod input is via AII amacrine cells. B) The mammalian retina functions over a very wide range of light intensities. Rods mediate responses under low light (scotopic) conditions, and as light levels increase cones and then melanopsin may contribute as rods become saturated. As an approximation, 1 lx of white light will be equivalent to roughly 12 log quanta, depending on the spectral power distribution of the light source. Figures based on (Lucas et al., 2014) (A) and (Dacey et al., 2005) (B).

As well as circadian entrainment and suppression of pineal melatonin in response to light, other biological responses were shown to be preserved in mice lacking rods and cones. Studies on the pupillary light response showed that not only were responses maintained in rodless, coneless mice, but the spectral sensitivity of these responses peaked around 478 nm, differing from the known visual pigments of the mouse retina (Lucas et al., 2001). In the late 1990s, several novel opsin photopigments had been identified in vertebrates, and one of these, melanopsin (OPN4), was found to be expressed in a subset of RGCs (Provencio et al., 1998, Provencio et al., 2000). These cells were shown to be directly photosensitive and projected to the hypothalamic suprachiasmatic nuclei (SCN), the site of the master circadian oscillator in mammals (Hattar et al., 2002). In retina from mice lacking rods and cones, or when rod and cones signals were pharmacologically blocked in rats, these photosensitive retinal ganglion cells (pRGCs) still responded to light (Berson et al., 2002, Sekaran et al., 2003). However, circadian and pupillary responses to light were only attenuated rather than abolished in melanopsin deficient mice (Lucas et al., 2003, Panda et al., 2002, Ruby et al., 2002). These findings demonstrated that whilst pRGCs were capable of mediating non-visual responses in the absence of rods and cones, rods and cones normally also contribute to these responses. Only when rods, cones and melanopsin were lost were all responses to light abolished (Hattar et al., 2003, Panda et al., 2003). Whilst pRGC responses were melanopsin-dependent, the final proof that melanopsin forms a functional photopigment came from studies using heterologous expression in cell lines that normally showed no light responses. Expression of melanopsin was sufficient to render these cells light-sensitive (Melyan et al., 2005, Panda et al., 2005, Qiu et al., 2005). Whilst rods and cones can mediate circadian responses in the absence of melanopsin, melanopsin-expressing pRGCs appear to provide the primary conduit for light information to non-visual responses to light. Targeted ablation of these cells abolished all circadian responses, even though rod and cone mediated vision was unaffected (Guler et al., 2008). As such, in the intact retina, light input from rods and cones and melanopsin are integrated at the level of the pRGCs (Fig. 3A). As well as their differences in spectral sensitivity, the role of rods, cones and melanopsin also depends upon the level of environmental irradiance. Rods mediate responses at very dim ‘scotopic’ light levels, but can also contribute under brighter ‘photopic’ conditions where visual function is dominated by cones (Altimus et al., 2010, Lall et al., 2010). Cones rapidly adapt and normally make a limited contribution under photopic conditions, where melanopsin plays a key role (Fig. 3B). However, they may contribute under conditions of high temporal contrast (Lall et al., 2010). Further complexity comes from the fact that different subtypes of pRGC have also been characterised, and these cells differ in their cellular anatomy, level of melanopsin expression, responses to light and projections to the brain (reviewed in (Hughes et al., 2016). Finally, melanopsin is expressed as either a long or short isoform, and these isoforms also show a different expression pattern and appear to be involved in different non-visual responses (Jagannath et al., 2015, Pires et al., 2009).

## 4 Non-image forming responses to light

**Fig. 4:**
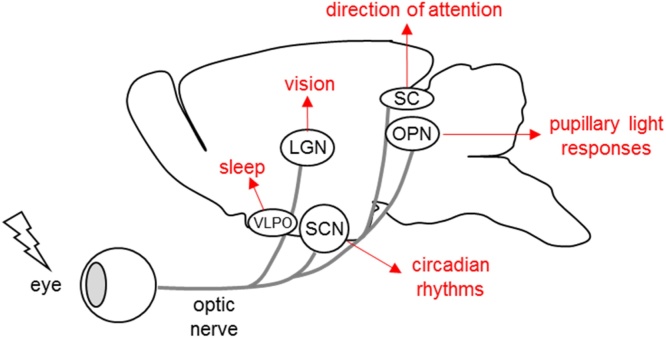
Summary of mouse visual and non-visual responses to light. Light detected by the retina is transmitted to the lateral geniculate nucleus (LGN) from which it is relayed to the visual cortex to mediate visual responses. By contrast, projections via the retinohypothalamic tract to the suprachiasmatic nuclei (SCN) mediate entrainment of circadian rhythms to light. Projections from melanopsin pRGCs to the olivary pretectal nucleus (OPN) mediate pupillary light responses, whereas projections to the ventrolateral preoptic nuclei (VLPO) modulate sleep. The superior colliculus (SC) receive input from both visual and non-visual pathways to direct attention to visual stimuli. Figure based upon (Hattar et al., 2006).

Characterisation of melanopsin pRGCs has led to the identification of a wide range of other physiological and behavioural responses to light. These are often termed non-image forming (NIF) or non-visual responses. Melanopsin pRGCs show diverse projections throughout the brain, differing from the classical visual pathways (Hattar et al., 2006). As well as the circadian entrainment and pupillary light responses described above, pineal melatonin suppression (Lucas et al., 1999), acute activity suppression (Mrosovksy and Hattar, 2003), sleep (Altimus et al., 2008, Lupi et al., 2008, Muindi et al., 2013, Pilorz et al., 2016, Tsai et al., 2009), corticosterone (Ishida et al., 2005, Pilorz et al., 2016), mood and cognition (LeGates et al., 2012, Tam et al., 2016) and even adaptation of visual pathways (Allen et al., 2014) have all been shown to be regulated by light. The classical visual pathways involve projections to lateral geniculate nucleus (LGN) and visual cortex, as well as direction of attention and gaze via light input to the superior colliculus (SC). Melanopsin pRGCs provide a major input to the master circadian pacemaker in the suprachiasmatic nuclei (SCN), but also project to the olivary pretectal nucleus (OPN) mediating pupillary light responses, and the ventrolateral preoptic nucleus (VLPO) which plays a key role in the regulation of sleep. These cells also innervate the SC, though the relative contributions of rods, cones and pRGCs to collicular responses remains largely unknown. Finally, the regulation of pineal melatonin suppression and corticosterone release are regulated via the SCN output to the sympathetic nervous system. The key retinal projections and the physiological and behavioural responses they mediate are summarised in Fig. 4.

## 5 Defects in visual and circadian function in inbred mouse lines

Studies on the visual and non-visual systems have provided much greater understanding of how retinal photoreceptors regulate diverse biological responses to light in mice. This work has also highlighted numerous different mutations that exist in commonly used inbred mouse strains that may affect these responses. The visual and non-visual function of a range of commonly used mouse strains are summarised in Table 1. These defects are described in more detail below.

**Table 1:** Visual and non-visual function in commonly used inbred mouse lines. Jackson lab references are provided to link to additional phenotypic data (www.jax.org). Retinal degeneration may be due to either.

Strain	JAX ref	Visual function	Non-visual function
129S1/SvImJ	002448	Normal	Melatonin deficient Low activity
A/J	000646	Albino	Melatonin deficient
BALB/cJ	000651	Albino	Melatonin deficient Short period
C3H/HeJ	000659	Retinal degeneration^a^	Melatonin proficient
C57BL/6J	000664	Normal	Melatonin deficient
C57BL/6N	005304	Retinal degeneration^b^	Melatonin deficient
CBA/J	000656	Retinal degeneration^a^	Melatonin proficient
DBA/2J	000671	Glaucoma >9 m	Melatonin deficient
FVB/NJ	001800	Retinal degeneration^a^	

a *Pde6b^rd1^* mutation.

b *Crb1^rd8^* mutation.

### 5.1 Albinism

Perhaps the most familiar mutation in inbred mouse lines is albinism, resulting from mutations in tyrosine 3-monooxegenase (tyrosinase), the enzyme which synthesises melanin from tyrosine in the skin and eyes (Jeffery, 1997). As well as the familiar white coat colour, tyrosinase mutations result in a deficiency of melanin in the retinal pigment epithelium (RPE), giving rise to a pink eye colouration. As the RPE normally absorbs light passing through the retina to prevent scatter, albino mice are also more sensitive to light and show impaired visual function. Albino mice are also more susceptible to light-induced retinal damage (De Vera Mudry et al., 2013). Melanin in the RPE also influences the developing neural retina. As such, albinos show reduced rod photoreceptor numbers, thinner inner and outer nuclear layers and reduced cell density in the retinal ganglion cell layer. Furthermore, during development, the routing of fibres in the optic tract is affected, leading to defects in the optic chiasm (Jeffery, 1997). Due to these complications, the study of visual or non-visual responses in albino inbred mouse lines is typically avoided.

### 5.2 Retinal degeneration 1 (*Pde6b^rd1^*)

Numerous forms of retinal degeneration (*rd*) have been characterised in the mouse (Chang et al., 2002, Chang et al., 2005). The original *rd* mutation was first characterised in several common laboratory inbred lines (Keeler, 1966, Pittler et al., 1993). Mice homozygous for the *rd1* mutation (*rd/rd*) have an early onset retinal degeneration in which the rods degenerate and cones are also subsequently lost, resulting in loss of visual responses. This is caused by a nonsense mutation in *Pdeb6*–the rod-specific phosphodiesterase that is a key component of the phototransduction cascade (Drager and Hubel, 1978, Pittler and Baehr, 1991). A second viral insert has accompanied this nonsense mutation, providing an alternative method of genotyping for the *rd1* mutation (Bowes et al., 1993).

### 5.3 Retinal degeneration 8 mutation (*Crb1*^*rd8*^)

Whilst numerous different retinal degeneration mutants have been identified, the *rd8* mutation is noteworthy due to its presence in many vendor strains of C57BL/6N mice (Mattapallil et al., 2012). Mice homozygous for *rd8* show a slow degeneration, accompanied by large white retinal deposits. At a histological level, these deposits correspond to areas of retinal folding. The *rd8* phenotype arises from a mutation in the *Crb1* gene, a homologue of the Drosophila *crumbs* gene (Mehalow et al., 2003), which appears to be important for cell polarity and maintaining the stratification of the retina (Bulgakova and Knust, 2009).

### 5.4 *Nob5* mutation (*Gpr179^nob5^*)

Another visual mutation that has been detected in common strains of laboratory mice is the *nob5* mutation, which arose spontaneously in a colony of C3H mice lacking the *Pde6b^rd1^* mutation. This mutation results in no-b wave (*nob*) in the electroretinogram (ERG), indicative of a defect in rod − bipolar cell signalling. The *nob5* mutation is caused by a DNA insertion in the orphan g-protein coupled receptor *Gpr179*. In humans, *Gpr179* mutations give rise to congenital stationary night blindness, where deficits in vision occur under rod-mediated scotopic light conditions (Peachey et al., 2012). This mutation has been detected in some C3H substrains, even leading to visual defects even where the more common *Pde6b^rd1^* mutation is absent (Balmer et al., 2013, Nishiguchi et al., 2015). However, a recent study has suggested that this mutation is not as widely distributed in vendor strains as has previously been suggested (Chang, 2015).

### 5.5 Melatonin deficiency

Melatonin is a hormone produced during the dark phase by the mammalian pineal. It is synthesised from serotonin primarily regulated by N-acetylserotonin (NAT) and hydroxyindole-o-methyltransferase (HIOMT). As well as been implicated in the regulation of circadian rhythms, in seasonally breeding animals, melatonin plays a key role in the photoperiodic response to day length (Pevet, 2003). Several inbred strains of laboratory mice, including both BALB/c and C57BL/6J lines, were found to not produce pineal melatonin due to two independent mutations in NAT and HIOMT (Ebihara et al., 1986). Subsequent studies identified that most inbred mouse lines are melatonin deficient, with the exception of C3H/He and CBA strains (Goto et al., 1989). More recently, a point mutation in NAT (Roseboom et al., 1998) and a high degree of polymorphism in the HIOMT gene (Kasahara et al., 2010) have been identified as the origin of melatonin deficiency in these lines.

### 5.6 Circadian behaviour

Differences in locomotor behaviour have been characterised between different mouse strains. These include both levels of voluntary wheel-running activity to differences in circadian period. Studies of home cage activity measured using beam-breaks has shown that C57BL/6J mice show higher activity than 129S1/SvImJ mice, particularly at the onset of the dark phase. F1 hybrids showed intermediate levels of activity (Hossain et al., 2004). Analysis of voluntary wheel-running activity in BALB/c, C57BL/6J and DBA mice showed differences in free-running period under constant darkness. DBA mice have a shorter circadian period of 23.31 h compared with 23.73 h in C57BL/6 mice, and BALB/c mice had an even shorter period of 22.9 h. Hybrid crosses were found to have approximately intermediate period values (Possidente and Stephan, 1988). These data are consistent with subsequent studies on 12 different inbred lines, which showed a range of periods from 22.94 h (BALB/c) up to 23.93 h (129/J) (Schwartz and Zimmerman, 1990). In addition to the level of activity and period, the variability of circadian period may also be more variable in some strains such as BALB/cJ (Siepka and Takahashi, 2005). The broad distribution of circadian period suggests that numerous alleles at different loci interact with the environment to generate the overt circadian period. This has led to several studies using quantitative trait locus (QTL) analysis to identify loci associated with circadian period and activity level (Hofstetter et al., 2003, Shimomura et al., 2001, Suzuki et al., 2001). More recently, studies using automated home cage analysis has allowed activity to be measured in cohoused mice which may enable a greater understanding of how activity of conspecifics affects circadian parameters (Bains et al., 2016). Finally, it should be noted that aging also affects circadian activity in a strain-dependent manner. A recent study identified changes in activity levels, period and many other circadian parameters which varied by background strain (Banks et al., 2015).

### 5.7 Sleep

Differences in sleep parameters between different mouse strains have also been extensively characterised. Differences is time spent awake and in REM and NREM sleep occur between inbred lines, as well as differences in EEG spectra during these different vigilance states (Franken et al., 1998, Franken et al., 1999). As described above, QTL analysis has exploited these strain-dependent differences in sleep to identify loci associated with different sleep-related parameters (Nicod et al., 2016, Tafti et al., 1999, Winrow et al., 2009). And as with circadian rhythms, aging also affects sleep in a strain-dependent manner (Banks et al., 2015, Hasan et al., 2012).

## 6 Practical considerations for rodent husbandry

Understanding the biological mechanisms mediating visual and non-visual responses to light critically underpins any evidence-based guidelines for the husbandry and welfare of laboratory mice. Here we provide some considerations for both researchers and animal facility staff based upon the known effects of light on mouse physiology and behaviour.

### 6.1 Measuring light in rodent research

Many studies do not provide any information relating to animal facility light levels − other than that they conform to local regulations. A problem with such regulations is that they are typically based upon the level of light required by the staff to work under, rather than any consideration of animal physiology. Where light measurements are provided, these are almost invariably reported in lux. A problem with such measurements is that lux is a unit based on perceived brightness weighted to the sensitivity of the human visual system. Whilst ideal for measuring the light environment for humans to work under, this is clearly not relevant to non-human species which possess a different spectral sensitivity to light. Lux is based upon the photopic sensitivity curve, which has a peak around 555 nm reflecting the red and green cones of the human retina. This is also not relevant under scotopic conditions under which rods provide the primary response, and even under photopic conditions, does not provide any reflection of melanopsin contributions to non-visual responses. Instead of using such photopic units, radiometric units based upon unweighted power measurements (e.g. μW/cm^2^/s) are more relevant (Foster et al., 2007, Peirson et al., 2005). Given that the interaction of light and any biological system involves the absorption of quanta of light, those working in the area of photobiology typically use measures based on photon flux. This is typically measured in photons/cm^2^/s, or the logarithm of this measurement − log quanta/cm^2^/s. As the power of a photon is inversely related to wavelength (i.e. short wavelength photons have higher energy), this means that given stimuli of equal power, a short wavelength stimuli will consist of fewer photons than one of longer wavelength. For this reason, when comparing the effects of wavelength, stimuli of equal photon flux should be used (isoquantal stimuli).

The primary reason for the use of lux in animal studies is that lux meters are cheap and readily available, and lux is the primary output of most commercially available light meters. By contrast, power meters and spectrophotometers (capable of measuring the spectral output of a light source) are considerably more expensive. Recent guidelines on the use of light in non-visual studies recommended that the spectral power distribution of all light sources should always be reported in such research to ensure reproducibility (Lucas et al., 2014). Whilst it is unrealistic to expect all facilities and researchers working with animals to adopt such strict guidelines, reporting lux measurements is better than no measurement. However, it is recommended that the type of lighting (e.g. fluorescent, incandescent or cool-white LED) is provided so that radiometric approximations can be made. A rodent irradiance toolbox was provided for this purpose, which also enables calculation of lux-derived units based on the photopigments of the mouse retina (freely available from: www.ndcn.ox.ac.uk/team/stuart-peirson).

### 6.2 Photoperiod

Mouse animal facilities house mice under 12:12 light/dark cycles (Jennings et al., 1998). Laboratory mice do not show seasonal changes in reproductive status in response to day length (Nelson and Zucker, 1981, Prendergast et al., 2001), which has been suggested to reflect their lack of pineal melatonin production (see above). However, even in melatonin proficient strains, there is no evidence of responses to day length, suggesting that the reason for this change is downstream of the pineal (Prendergast et al., 2001). However, photoperiod may affect circadian physiology as day length has been shown to affect both cellular coupling in the SCN pacemaker and behavioural outputs in laboratory mice (Coomans et al., 2015).

### 6.3 Reversing light/dark cycles

The circadian system drives profound changes in physiology and behaviour over the 24 h cycle. As such, normal physiology is highly dynamic, with constant variation around homeostatic set-points (Mrosovsky, 1990). For example, a recent study of the transcriptome of 12 different mouse organs found that 43% of protein encoding genes showed circadian rhythms in transcription somewhere in the body − typically in a tissue-dependent manner. Moreover, many of the most widely used drugs target the products of these rhythmic genes (Zhang et al., 2014). This clearly poses a problem for any biological measurement (Burns, 2000). The aim of using mice in research is usually to attempt to understand human physiology and disease. However, as a nocturnal species, most studies on mice are conducted during the light period when they are typically inactive or asleep. This has led to the obvious suggestion that studies on laboratory mice will be more ethologically relevant and yield more reliable data if conducted during the dark phase which mice are normally awake and active (Peirson and Foster, 2011). In addition, it has been suggested that data from nocturnal mice will more closely correspond to a daytime human (McLennan and Taylor-Jeffs, 2004). The most obvious way to achieve this would be to reverse the light dark cycle in the animal facility so that mice can be tested in the dark during the human working day.

There are limited data on whether dark phase testing improves experimental outcomes. The ability to discriminate differences between genetically distinct mice has been shown to be improved when performed in the dark phase, but only on the SHIRPA behavioural test battery, open field and rotarod tests. However, many other tests were unaffected, and the tail flick test showed improved discrimination in the light phase (Hossain et al., 2004). Studies on DBA mice have shown that testing during the light phase results in behavioural inhibition and cognitive impairment (Roedel et al., 2006). However, other studies have found either no effect (Beeler et al., 2006) or actually improved performance during the subjective day (Chaudhury and Colwell, 2002). The latter is particularly relevant, involving the use of fear conditioning to study acquisition, recall and extinction. Mice acquired the conditioning faster when trained during the day, and recall peaked during the day, irrespective of time of training. Extinction was greater in mice trained at night. Finally, all three rhythms persisted in constant conditions, demonstrating that these were circadian rhythms and not just driven by the light/dark cycle (Chaudhury and Colwell, 2002). Moreover, data from a within-subjects study in our own laboratory have shown that object recognition performance in mice is similarly better during the light phase than the dark phase, and this pattern persists under constant conditions (Tam et al., 2017). Why learning and memory should be better during a nocturnal animal’s normally inactive phase is unclear, but may relate to the effects of preceding sleep and arousal or differences in response to handling.

An additional consideration for testing mice during their dark phase are circadian rhythms in retinal function. The photopic electroretinogram (ERG) shows circadian rhythms in b-wave amplitude, with reduced amplitude responses in the dark phase (Cameron et al., 2008). Similarly, contrast sensitivity, the ability to detect an object against background, peaks during the daytime and is reduced during the night (Hwang et al., 2013). It is perhaps not surprising that the retina has evolved to upregulate these pathways during the day when they will be required in the light, and downregulating them in darkness. As such, any test depending upon visual cues performed during the animal’s active phase should consider that visual function may be impaired.

An obvious consideration of testing mice in the dark relates the simple operational issue of how do the facility staff and scientific researchers operate under such conditions? This requires those working in the facility to either use infrared night vision devices or operate under very dim light (see below). Under such conditions, welfare monitoring is considerably more difficult, and early indicators of illness or adverse effects following procedures may potentially be missed. Similarly, problems with housing, such as leaking water bottles or poorly fitting cage lids may be overlooked, and the time taken to perform routine husbandry duties is considerably increased. In addition, operating under dim light raises obvious additional health and safety concerns. A final consideration is that reversing the light/dark cycle will result in the animal’s active phase coinciding with when the animal facility is at its busiest. We do not know the consequences of such a change, but given that mice and humans have coexisted for millennia occupying different temporal niches, forcing mice to be active during the human working day could be a potentially unexpected stressor. As the data on behavioural testing and visual function described above demonstrate, studying a nocturnal species at night is not the same as studying a diurnal species during the day, and overlooks many other differences in physiology and behaviour.

### 6.4 Mice respond to red light

An influential report published by the Rodent Refinement Working Party included the statement ‘*Mice are insensitive to red light so this is useful for observation purposes*’ (Jennings et al., 1998). Whilst partially correct − mice are indeed less sensitive to red light − this has been taken by many to suggest that mice simply do not respond to red light. This is clearly untrue − numerous irradiance response curves to long wavelength light have been published, and whilst less sensitive, responses to bright stimuli occur (Butler and Silver, 2011, Hattar et al., 2003, Lucas et al., 2001). As a result of this misunderstanding of photobiology, it has been suggested that a simple solution for the problem of reversing light/dark cycles in animal facilities is to use red light (typically >600 nm) or sodium light (with a peak emission around 589–590 nm). Such conditions would allow humans to see, but would be ‘on the margins of mouse visual sensitivity’ (McLennan and Taylor-Jeffs, 2004). However, based on the known visual pigments of the mouse retina, mice are around 12 time less sensitive than humans to 600 nm red light, and around 8 times less sensitive to 589 nm sodium light. Available data for various non-visual biological responses in mice have shown that the threshold sensitivity of these responses is very low (Table 2). As such, the level of nocturnal light required for humans to operate would almost certainly produce biological responses in mice. Indeed, where sodium lighting has been used, light levels within the cage ranged from 9.6 lx at the top of a cage rack down to 1.2 lx at the bottom. Light levels outside the cage were substantially higher (McLennan and Taylor-Jeffs, 2004). To place this in context, within the cage, this would correspond to 12.7 down to 11.8 log quanta/cm^2^/s. Based on the mouse MWS cone with a λ_max_ at 508 nm, mice are just 1 log unit less sensitive at 589–90 nm compared with peak sensitivity. As can be seen from the data shown in Table 2, this would suggest that except for lower cage positions, such levels of sodium lighting are above the threshold required to evoke biological responses in mice.

**Table 2:** Threshold sensitivity of laboratory rodents for different biological responses to light. Values are provided in log quanta/cm^2^/s. Where thresholds are not specifically determined, these were derived from irradiance response curves as the approximate photon flux required to elicit a 10% response. Data are shown for peak sensitivity or white light, as well as for long wavelength light (LWL). Peak wavelengths are noted in parenthesis (White = white light, typically fluorescent). References for original studies are provided along with mouse strain. Where data are provided in photopic units or power, quantal values were approximated using the freely-available irradiance toolbox (Lucas et al., 2014).

Response	Threshold logQ (λ)	Threshold logQ (LWL)	Strain and reference
Circadian entrainment and phase shifting	9.1 (518 nm)	11.9 (635 nm)	C57 (Butler and Silver, 2011)
	9.9 (White)	–	C57 (Ebihara and Tsuji, 1980)
	11.9 (White)	–	C3H (Ebihara and Tsuji, 1980)
	11.9 (White)	–	C3H^a^ (Foster and Helfrich-Forster, 2001)
	9.9 (515 nm)	–	C3H^a^ (Foster et al., 1991)
	10.5 (500 nm)	12.5 (600 nm)	CBA (Yoshimura and Ebihara, 1996)
	9.4 (509 nm)	–	C3H^a^ (Freedman et al., 1999)
	10.5 (506 nm)	11.5 (580 nm)	C3H^b^ (Hattar et al., 2003)

Pupillary light response	9.0 (506 nm)	10.5 (625 nm)	C3H^a^ (Lucas et al., 2001)
	9.0 (480 nm)	–	Lucas et al. (2003)
	10.5 (470 nm)	–	Van Gelder et al. (2003)
	11.4 (517 nm)	12.4 (635 nm)	Butler and Silver, (2011)

Negative masking	12.0 (518 nm)	–	C57 (Butler and Silver, 2011)
	11.6 (505 nm)	–	C57 (Thompson et al., 2008)
	12.5 (505 nm)	–	129 (Thompson et al., 2008)
	12.6 (White)	–	C57 (Mrosovsky et al., 1999)
	12.9 (White)	–	C3H (Mrosovsky et al., 1999)
	13.7 (White)	–	CBA (Mrosovsky et al., 1999)

Melatonin suppression	9.4 (509 nm)	–	C3H (Lucas et al., 1999)
	9.9 (503 nm)	–	Hamster^c^ (Nelson and Takahashi, 1991)
	10.7 (500 nm)	–	Hamster^c^ (Podolin et al., 1987)
Corticosterone induction	12.9 (White)	–	C57 (Ishida et al., 2005)

a Data from C3H mice not carrying the *Pde6b^rd1^* mutation.

b Data from *rd/rd cl* mice, lacking all rods and cones.

c Data from hamster due to melatonin deficiency in many common laboratory mouse lines.

Numerous studies have been performed on light/dim light cycles, with the aim of studying how light at night affects physiology and behaviour. Whilst mice appear to entrain under such conditions, confining their activity to the dim light phase, these studies have shown a wide range of adverse effects, including changes in weight, metabolism, immune function, cognition, anxiety and affective responses (Bedrosian et al., 2013, Fonken et al., 2009, Fonken and Nelson, 2014, Fonken et al., 2013, Navara and Nelson, 2007). Any initiative to reverse light/dark cycles involving use of long wavelength light during the animal’s active phase must consider the intensity of such lighting. If sufficiently bright to produce biological responses, long term housing under such conditions may be equivalent to the light at night protocols described above, and may have a greater welfare cost than first envisaged.

### 6.5 Sensitivity thresholds for responses to light

Given the number of studies that have investigated the spectral sensitivity of non-visual responses it is possible to provide some guidance of the thresholds of different biological responses to light. A range of studies are summarised in Table 2, including responses around peak sensitivity (typically around 470–520 nm) and to longer wavelengths (typically 580nm–635 nm). As can be seen, the thresholds for circadian entrainment, pupillary light responses and melatonin suppression are in the 9–10 log quanta range at peak. To place this into context, 1 lx of white light will be equivalent to roughly 12 log quanta (depending on the spectral power distribution of the light source), meaning that 0.01 lx may be sufficient to evoke a biological response. Based the available data, thresholds for responses to long wavelength stimuli are typically 1–2 log units less sensitive than at peak, which is consistent with the sensitivity of the mouse retina at these wavelengths. For example, based on the sensitivity of the mouse MWS cone opsin, which is the most long-wavelength sensitive of the mouse retinal photopigments, at 580 nm, absorption is around 17% (−0.8 log units), at 600 nm absorption is around 5% (−1.3 log units) and at 635 nm absorption is around 0.4% (−2.4 log units) (Govardovskii et al., 2000, Sun et al., 1997). As such, in the absence of specific studies addressing these issues, aiming for <1 lx of 600 nm red light (<11.8 log quanta/cm^2^/s) would be a practical starting point for brief observation to minimise the effects on non-visual processes. However, as shown in Fig. 3B, due to the sensitivity of rod photoreceptors to very dim light, visual sensitivity may extend well below this level.

## 7 Conclusions

Light exerts widespread effects on the physiology and behaviour of all mammals, and mice are no exception. As well as the classical rod and cone visual pathways, recent research has identified an entirely novel retinal photoreceptive system, consisting of a subset of retinal ganglion cells expressing the photopigment melanopsin. These cells are involved in the regulation of a wide range of non-visual responses to light including entrainment of the circadian clock, pupillary constriction, regulation of hormones such as melatonin and corticosterone and even the modulation of sleep and cognition. Understanding the effects of light on mouse physiology and behaviour must take into account how these different photoreceptor systems function, including their differing sensitivities to both level and wavelength of light, as well as how they interact. In addition, a wide range of mutations that affect visual and non-visual processes exist in commonly used inbred mouse strains. These must be taken into consideration when studying the effects of light on laboratory mice. It has been suggested that reversing light/dark cycles in the animal facility may provide an obvious way to study mice during their nocturnal active phase, which may extrapolate better to human diurnal physiology. However, studying a nocturnal species at night is not the same as studying a diurnal species during the day, and adoption of such conditions must recognise these differences in temporal biology and consider the potential unintended consequences. For example, circadian rhythms in learning and memory and visual function in mice mean that performance in the nocturnal active phase may actually be impaired. Finally, whilst mice are less sensitive than humans to red light, they are still capable of responding to long wavelength stimuli. As such, studies on mice conducted during the dark phase must consider both the intensity as well as the wavelength of the light sources used. A greater appreciation of the effects of the light environment is essential for the reproducibility of scientific studies as well as for refining the housing conditions of laboratory mice.
